# Differential effects of aerobic, resistance, and combined exercise on skeletal muscle function in high-fat diet–induced obese mice: integration of gut microbiota and metabolomics

**DOI:** 10.3389/fendo.2026.1817173

**Published:** 2026-06-12

**Authors:** Yefan Wang, Pengpeng Gao, Zijun Hao, Jinjing Cui, Faying Song, Lijun Wu

**Affiliations:** School of Physical Education, Shanxi University, Taiyuan, China

**Keywords:** exercise intervention, gut microbiota, high-fat diet-induced obesity, metabolomics, skeletal muscle function

## Abstract

**Objective:**

This study tested the hypothesis that aerobic, resistance, and combined exercise differentially remodel gut microbiota composition and serum metabolic profiles, and that these modifications are associated with modality-specific improvements in skeletal muscle function and morphology through the gut–muscle axis in high-fat diet–induced obese mice.

**Methods:**

Six-week-old male C57BL/6J mice were randomly assigned to a control group (C, standard diet), a high-fat diet group (HF), and high-fat diet groups subjected to aerobic exercise (HFA), resistance exercise (HFR), or combined exercise (HFAR). Exercise interventions were conducted for 8 weeks. Following the intervention, skeletal muscle endurance and strength were assessed. Tibialis anterior muscles were harvested for hematoxylin–eosin, Oil Red O, Picro-Sirius Red, and succinate dehydrogenase staining. Mitochondrial DNA (mtDNA) copy number was quantified as a marker of mitochondrial biogenesis. Gut microbiota composition was analyzed by 16S rRNA gene sequencing, and serum metabolites were profiled using untargeted liquid chromatography–mass spectrometry metabolomics.

**Results:**

Compared with the HF group, all exercise groups showed improvements in treadmill endurance and relative grip strength, along with amelioration of the pathological morphological alterations observed in skeletal muscle, including reduced lipid accumulation and fibrosis. The HFAR exhibited the most pronounced effects, including improved muscle fiber morphology, an increased proportion of slow-twitch fibers, elevated mtDNA content, and reduced lipid deposition and fibrosis. Gut microbiota analysis demonstrated significantly reduced α-diversity in the HF group compared to the C group, whereas the HFAR group presented the most substantial restoration of diversity. β-diversity analysis indicated that exercise interventions shifted gut microbiota composition toward that of the control group. Linear discriminant analysis effect size analysis identified exercise modality–specific microbial changes, including enrichment of *Blautia* in the HFR group and *Romboutsia* in the HFAR group. Metabolomic analysis identified 278 differential metabolites, with distinct regulatory patterns across metabolic pathways depending on exercise modality. Correlation analysis demonstrated significant associations between specific bacterial genera and differential metabolites.

**Conclusions:**

Aerobic, resistance, and combined exercise differentially ameliorated skeletal muscle dysfunction in high-fat diet–induced obese mice. These effects were associated with modality-specific changes in gut microbiota composition and host metabolic profiles.

## 1 Introduction

Changes in contemporary dietary patterns and increasingly sedentary lifestyles have contributed to a substantial rise in the global prevalence of obesity over recent decades. This condition now affects more than one billion individuals and constitutes a major public health concern (1). Obesity is recognized as a disease that adversely affects quality of life, reduces life expectancy, and is directly associated with multiple chronic conditions (2). A high-fat diet, a principal contributor to obesity, promotes lipid accumulation within skeletal muscle, reduces muscle fiber diameter, and activates protein degradation pathways, thereby accelerating muscle atrophy (3). Inflammatory mediators derived from intermuscular adipose tissue suppress mitochondrial activity and impair metabolic flexibility. This is characterized by enhanced glycolysis and reduced oxidative capacity, which ultimately contributes to glucose intolerance and insulin resistance (4, 5).

Exercise has been demonstrated to exert beneficial effects on multiple complications associated with obesity, and different exercise modalities have demonstrated efficacy in reducing adiposity and improving body composition (6). With respect to skeletal muscle function, modality-specific effects have been reported. Aerobic exercise improves skeletal muscle function in individuals with obesity by enhancing metabolic capacity, including glucose and lipid metabolism, thereby increasing insulin sensitivity (7). In contrast, resistance training has demonstrated significant benefits in increasing skeletal muscle mass and cross-sectional area, as well as enhancing skeletal muscle strength (8). It is noteworthy that most existing studies have primarily examined the effects of a single exercise modality on obesity, whereas investigations assessing combined exercise (i.e., the concurrent integration of multiple exercise modalities) remain limited. The mechanisms through which aerobic exercise, resistance exercise, and combined exercise ameliorate obesity-induced skeletal muscle dysfunction have not yet been fully elucidated.

The gut microbiota plays a key role in the development of obesity and related diseases (9). In recent years, advances in this field have led to the proposal of the gut microbiota–muscle axis, highlighting the influence of gut microbiota on skeletal muscle structure and function (10). Exercise is widely recognized as an effective strategy for enhancing gut microbiota diversity and increasing the relative abundance of beneficial bacteria (11). However, limited evidence is available regarding whether different exercise modalities exert distinct effects on gut microbiota remodeling under HFD conditions and if such differences contribute to differential improvements in skeletal muscle function. Metabolomics, which enables comprehensive characterization of systemic metabolic changes following external interventions, serves as a key tool for elucidating gut microbiota–host interactions (12). Therefore, this study used 16S rRNA gene sequencing and liquid chromatography–mass spectrometry (LC-MS)-based metabolomics in a high-fat diet–induced obesity model to systematically compare how aerobic, resistance, and combined exercise differentially modulate gut microbiota composition and host systemic metabolic profiles, and to examine whether these differential microbial and metabolic signatures are associated with modality-specific improvements in skeletal muscle structure and function through the gut-muscle axis.

## 2 Materials and methods

### 2.1 Experimental animals and grouping

Six-week-old male C57BL/6J mice were obtained from Beijing Vital River Laboratory Animal Technology Co., Ltd. (License No.: SYXK (Hu) 2023-0005). Following one week of acclimatization, the mice were randomly assigned to either a standard chow group or a high-fat diet model group. The standard chow group was continuously provided with a national standard regular diet (3.85 kcal/g, with 24% protein, 12% fat, and 64% carbohydrate as a percentage of total calories), whereas the high-fat diet model group was fed a high-fat diet (XTM04-001, Jiangsu Xietong Pharmaceutical Bio-engineering Co., Ltd.; 5.24 kcal/g, with 20% protein, 60% fat, and 20% carbohydrate as a percentage of total calories) for 8 consecutive weeks to induce obesity. Successful model establishment was defined as a body weight in the high-fat diet group exceeding the average body weight of the standard chow group by ≥ 20% after 8 weeks of feeding (13). Mice that met the modeling criteria were subsequently randomly allocated into four groups: high-fat diet (HF) group, high-fat diet + aerobic exercise (HFA) group, high-fat diet + resistance exercise (HFR) group, and high-fat diet + combined exercise (HFAR) group, with six mice in each group. An additional six mice from the standard chow group were randomly selected to serve as the control (C) group. All mice were housed six per cage, under controlled environmental conditions (temperature 25–28 °C, relative humidity 40%–60%, and a 12 h/12 h light–dark cycle). The control group received the standard chow diet, whereas all other groups continued to receive the high-fat diet. All experimental procedures involving animals were conducted in accordance with the guidelines of the Committee of Scientific Research in Shanxi University and were approved under authorization number SXULL2025081.

### 2.2 Exercise intervention

The exercise intervention protocol was adapted and modified from previously published methods (14, 15). Prior to the formal intervention, all exercise groups completed a 6-day adaptation period. During this period, mice in the HFA group underwent treadmill running (JLBehv-TDMG, Shanghai Jiliang Software Science & Technology Co., Ltd.) at a speed of 9 m/min for 30 minutes per day. Mice in the HFR group performed ladder-climbing exercise without additional load, consisting of two sets per day with three repetitions per set. Mice in the HFAR group performed the two training modalities on alternating days.

Following the adaptation period, a one-day rest interval was provided prior to initiation of the 8-week formal intervention.

The formal training protocol was implemented as follows. Mice in the HFA group underwent treadmill training at a 0° incline. Exercise intensity and duration were progressively increased from 12 m/min for 30 minutes in week 1 to 15 m/min for 60 minutes by week 8. Mice in the HFR group performed loaded ladder-climbing exercise. The load was progressively increased from 10% of body weight in week 1 to 70% of body weight by week 8. Mice in the HFAR group performed aerobic treadmill training on Mondays, Wednesdays, and Fridays, and resistance ladder-climbing training on Tuesdays, Thursdays, and Saturdays. Body weight was measured weekly during the intervention period to allow appropriate adjustment of resistance training loads. The ladder specifications (Lad-V-M85, Beijing Cinontech Co., Ltd.) were as follows: height 1 m, width 20 cm, step height 1 cm, and inclination angle 85°. Detailed training parameters and the progression schedule are presented in **Table 1**.

**Table 1:** Formal training scheme.

Weeks	HFA	HFR	HFAR
Week 1	Treadmill speed: 12 m/min Training duration: 30 min	Load: 10% of body weight	Aerobic treadmill training on Mon, Wed, Fri. Loaded ladder climbing resistance training on Tue, Thu, Sat.
Weeks 2 & 3	Treadmill speed: 12 m/min Training duration: 45 min	Load: 30% of body weight	
Weeks 4 & 5	Treadmill speed: 15 m/min Training duration: 45 min	Load: 50% of body weight	
Weeks 6, 7 & 8	Treadmill speed: 15 m/min Training duration: 60 min	Load: 70% of body weight	

### 2.3 Skeletal muscle function tests

Skeletal muscle endurance test: Endurance capacity was assessed using a treadmill exhaustion protocol. The procedure consisted of a 5-minute warm-up at 5 m/min, followed by running at 10 m/min for 40 minutes. The speed subsequently increased by 1 m/min every 10 minutes for 30 minutes and then by 1 m/min every 5 minutes until exhaustion. Exhaustion was defined as remaining at the rear of the treadmill for 30 seconds or failure to respond to external stimulation. Time to exhaustion was recorded (16).

Forelimb grip strength test: Forelimb grip strength was measured using a grip strength meter (BIO-GS4, RWD Life Science). Five trials were conducted for each mouse, and the maximum value was recorded and normalized to body weight (N/g) (17).

### 2.4 Sample collection and processing

Mice were fasted for 24 hours prior to tissue collection to minimize the acute effects of food intake on serum metabolomic profiles and gut microbiota composition, and to establish a uniform metabolic baseline across all groups. Water was provided ad libitum during the fasting period. On the day of sample collection, body weight was recorded. General anesthesia was induced by isoflurane inhalation, and adequate anesthetic depth was confirmed by the absence of a plantar reflex. Blood samples were obtained from the retro-orbital venous plexus. The collected blood was allowed to clot at room temperature for 30 minutes and subsequently centrifuged at 3,000 rpm for 10 minutes to separate serum from cellular components. The supernatant serum was collected and stored for subsequent analysis. Following blood collection, euthanasia was performed by cervical dislocation. Immediately thereafter, the left tibialis anterior muscle, quadriceps muscle, adipose tissue, and colonic contents were harvested. Colonic contents were collected aseptically into sterile cryotubes, snap-frozen in liquid nitrogen, and stored at −80 °C until DNA extraction. Subcutaneous (inguinal), perirenal, and epididymal white adipose tissues were dissected and weighed. White adipose tissue percentage was calculated using the formula: white adipose tissue percentage = (subcutaneous fat [g] + perirenal fat [g] + epididymal fat [g])/terminal body weight [g] × 100% (18)The tibialis anterior and quadriceps muscles, as well as the adipose tissue, were gently blotted dry using filter paper, rapidly weighed, and snap-frozen in liquid nitrogen prior to storage at −80 °C. The tibialis anterior muscle was additionally embedded in optimal cutting temperature compound for frozen sectioning and subsequent immunohistochemical staining.

### 2.5 Mitochondrial DNA content detection

Mitochondrial DNA (mtDNA) copy number was quantified by measuring the expression of the mitochondrial gene *Nd1* (NADH dehydrogenase subunit 1) using reverse transcription quantitative polymerase chain reaction (RT-qPCR) (Forward: 5’-CACTATTCGGAGCTTTACG-3’; Reverse: 5’-TGTTTCTGCTAGGGTTGA-3’). The expression of the nuclear DNA–encoded gene *Lpl* (lipoprotein lipase) was measured to represent genomic DNA content (Forward: 5’-GAAAGGGCTCTGCCTGAGTT-3’; Reverse: 5’-TAGGGCATCTGAGAGCGAGT-3’). mtDNA content was calculated as the ratio of *Nd1* to *Lpl* (19).and served as a surrogate marker of mitochondrial biogenesis.

### 2.6 Tissue staining

Frozen sections (8–10 μm thick) of the tibialis anterior muscle were prepared and processed for histological analysis as follows: (1) Hematoxylin and eosin (HE) staining was conducted to assess skeletal muscle morphology; (2) Oil Red O staining was conducted to quantify the extent of ectopic lipid deposition within skeletal muscle tissue; (3) Picro-Sirius Red staining was used to assess the fibrotic area in skeletal muscle; and (4) succinate dehydrogenase (SDH) staining was conducted to determine the proportion of slow-twitch muscle fibers in skeletal muscle.

### 2.7 Gut content 16S rRNA sequencing

Total genomic DNA was extracted from intestinal content samples using the CTAB method. DNA quality was assessed by agarose gel electrophoresis and Qubit fluorometric quantification. The V3–V4 hypervariable region of the bacterial 16S rRNA gene was amplified by polymerase chain reaction using primers 341F and 806R. The amplification conditions were as follows: initial denaturation at 98 °C for 1 minute; 30 cycles of 98 °C for 10 seconds, 50 °C for 30 seconds, and 72 °C for 30 seconds; followed by a final extension at 72 °C for 5 minutes.

Sequencing libraries were prepared using the Illumina NovaSeq platform (NovaSeq Reagent Kit, 250 bp paired-end), and high-throughput sequencing was performed. Raw sequencing reads were subjected to quality control (QC) using fastp and paired-end reads were merged using FLASH software. Amplicon sequence variants (ASVs) were generated using the Deblur algorithm. Representative ASV sequences were taxonomically classified using the RDP classifier against the Silva database (v138).

All samples were rarefied to the minimum sequencing depth prior to downstream analysis. Alpha diversity indices (ACE, Chao1, Shannon, and Simpson) and beta diversity analyses [principal coordinates analysis (PCoA) based on Bray–Curtis and weighted UniFrac distances] were subsequently performed.

### 2.8 Serum untargeted LC-MS metabolomics

Serum samples were pretreated using a methanol precipitation method, and the resulting supernatant was collected for LC–MS analysis. Metabolite separation was performed using a Thermo Scientific Vanquish UHPLC system equipped with an ACQUITY Premier HSS T3 column. Mass spectrometric analysis was conducted on a Thermo Scientific Q Exactive HF-X mass spectrometer. Data were acquired in both positive and negative ionization modes using an electrospray ionization source.

Full-scan acquisition was performed at a resolution of 70,000 across a mass range of m/z 75–1000. Raw mass spectrometry data were subjected to standard preprocessing procedures, including peak extraction, retention time correction, and peak area normalization. Metabolite identification was conducted by comparison with public databases, including HMDB and KEGG. To assess analytical stability, QC samples were inserted throughout the analytical sequence. Differential metabolites were screened using an orthogonal partial least squares discriminant analysis (OPLS-DA) model. Selection criteria were defined as a variable importance in projection (VIP) score > 1.0 and *p* < 0.05 based on Student’s t-test.

### 2.9 Statistical analysis

Statistical analyses were conducted using SPSS version 26.0 and R software (v4.1.2). After Shapiro-Wilk and Levene’s tests, normally distributed data with equal variances were analyzed by one-way ANOVA with Tukey’s HSD *post hoc* test and presented as mean ± SEM. Data with unequal variances were analyzed by Welch’s ANOVA with Games-Howell *post hoc* test. Non-parametric data were analyzed by Kruskal-Wallis H test with Bonferroni-adjusted Mann-Whitney U test (adjusted α = 0.005) and presented as median (interquartile range). Effect sizes were reported as partial η² or ϵ². Associations among differential genera, metabolites, and phenotypic indices were assessed by Spearman rank correlation. Exploratory mediation analyses were conducted using the PROCESS macro (Model 4, 5000 bias-corrected bootstrap resamples), with differential genera as independent variables, differential metabolites as mediators, and phenotypic indices as dependent variables; mediation was supported if the 95% bootstrap CI for the indirect effect excluded zero. All mediation results are interpreted as hypothesis-generating.

## 3 Results

### 3.1 Effects of different exercise modalities on body weight, energy intake, body composition, and skeletal muscle function

**Figure 1:**
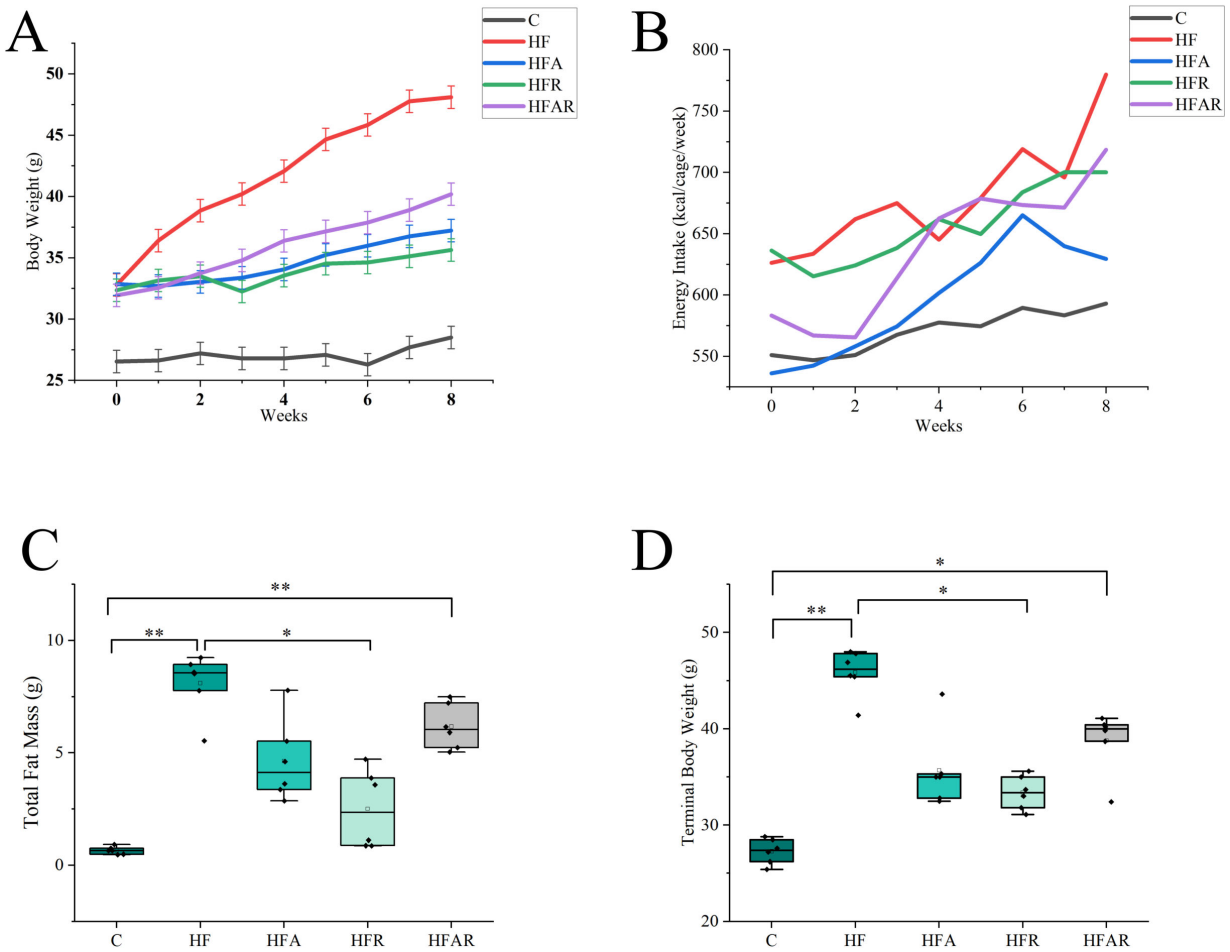
Effects of different exercise modalities on body weight, energy intake, terminal fat mass, and terminal body weight in HFD-fed mice. **(A)** Weekly body weight trajectories.; **(B)** Weekly energy intake recorded at the cage level (kcal/cage/week). Data are from a single cage per group (n = 6 mice/cage, 1 cage/group); **(C)** Terminal total fat mass, Data are median and interquartile range (n = 6/group), *p < 0.005, **p < 0.001; **(D)** Terminal body weight after 24-h fasting, measured immediately before sacrifice, Data are median and interquartile range (n = 6/group), *p < 0.005, **p < 0.001.

Weekly body weight trajectories across the 8-week intervention are presented in **Figure 1A**. The C group showed steady growth throughout, while the HF group continued to gain weight and remained the heaviest group at most time points. Among the exercise groups, HFR consistently exhibited the lowest body weight, followed by HFAR and HFA. Two-way repeated measures ANOVA revealed a significant Time × Group interaction (F = 37.445, p < 0.01). Weekly energy intake (**Figure 1B**, kcal/cage/week) is presented descriptively. The C group consumed the fewest calories throughout the experiment. Among HFD-fed groups, the HF group exhibited the highest energy intake at the endpoint, HFR maintained high intake throughout, HFA started with the lowest intake and progressively increased, and HFAR remained relatively stable. Due to cage-level recording (n = 1 cage per group), no inferential statistical tests were applied.

**Figure 2:**
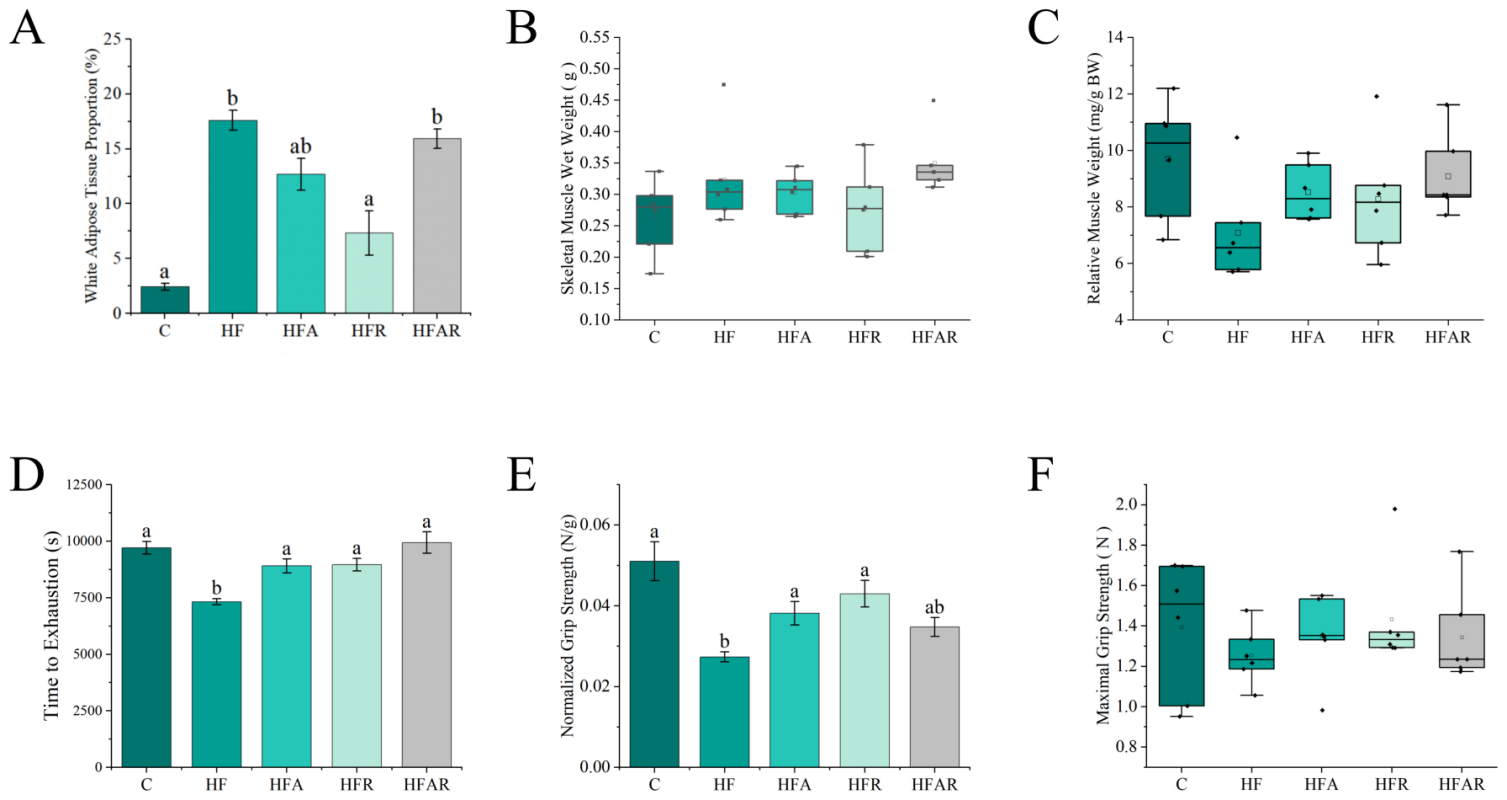
Effects of different exercise modalities on body composition, skeletal muscle weight, and functional indices in HFD-fed mice. **(A)** White adipose tissue percentage. **(B)** Skeletal muscle wet weight (tibialis anterior + quadriceps). **(C)** Relative muscle weight (muscle wet weight / terminal body weight). **(D)** Time to exhaustion. **(E)** Normalized grip strength (grip strength / terminal body weight). **(F)** Maximal grip strength. Column charts: Data are expressed as mean ± SEM (n = 6 per group), different letters indicate statistically significant differences among groups. Box plots: The box represents the interquartile range (IQR, 25th–75th percentiles).

Terminal total fat mass (**Figure 1C**) differed significantly among groups (Kruskal-Wallis H = 23.819, p < 0.001, ϵ² = 0.793). The HF and HFAR groups had significantly higher fat mass than the C group (p < 0.001), whereas HFR showed significantly lower fat mass compared with HF (p < 0.005). Terminal body weight displayed a similar pattern (**Figure 1D**, H = 23.924, p < 0.001, ϵ² = 0.797). White adipose tissue percentage further confirmed these trends (**Figure 2A**, Welch’s F(4, 12.34) = 96.161, p < 0.001, ω² = 0.930). The HF group exhibited significantly higher values than the C group (p < 0.001). Only HFR showed a significant reduction compared with HF (p = 0.014), whereas HFA (p = 0.004) and HFAR (p < 0.001) remained significantly elevated relative to C.

Absolute wet weight of the left hindlimb (tibialis anterior + quadriceps) did not differ significantly among groups (**Figure 2B**, H = 8.288, p = 0.082, ϵ² = 0.286). Numerically, the HF group did not show a deficit relative to the C group. After normalization to terminal body weight, relative muscle weight (**Figure 2C**) also did not reach significance (H = 7.720, p = 0.102, ϵ² = 0.266), but all three exercise groups trended toward the C-group level. Treadmill exhaustion time (**Figure 2D**) was significantly shorter in the HF group compared with C (p < 0.01) and all exercise groups (p < 0.05, partial η² = 0.632). Absolute grip strength did not differ among groups (**Figure 2E**, H = 2.564, p = 0.633, ϵ² = 0.088). In contrast, relative grip strength showed significant group differences (**Figure 2F**, Welch’s F(4, 12.46) = 16.886, p < 0.001, ω² = 0.629), with the HF group significantly lower than C, HFA, and HFR (p < 0.005). This dissociation between absolute and relative grip strength indicates that force output per unit body mass was significantly impaired under HFD conditions and was attenuated by exercise, particularly HFA and HFR, without a concomitant change in absolute force.

### 3.2 Effects on skeletal muscle histomorphology

**Figure 3:**
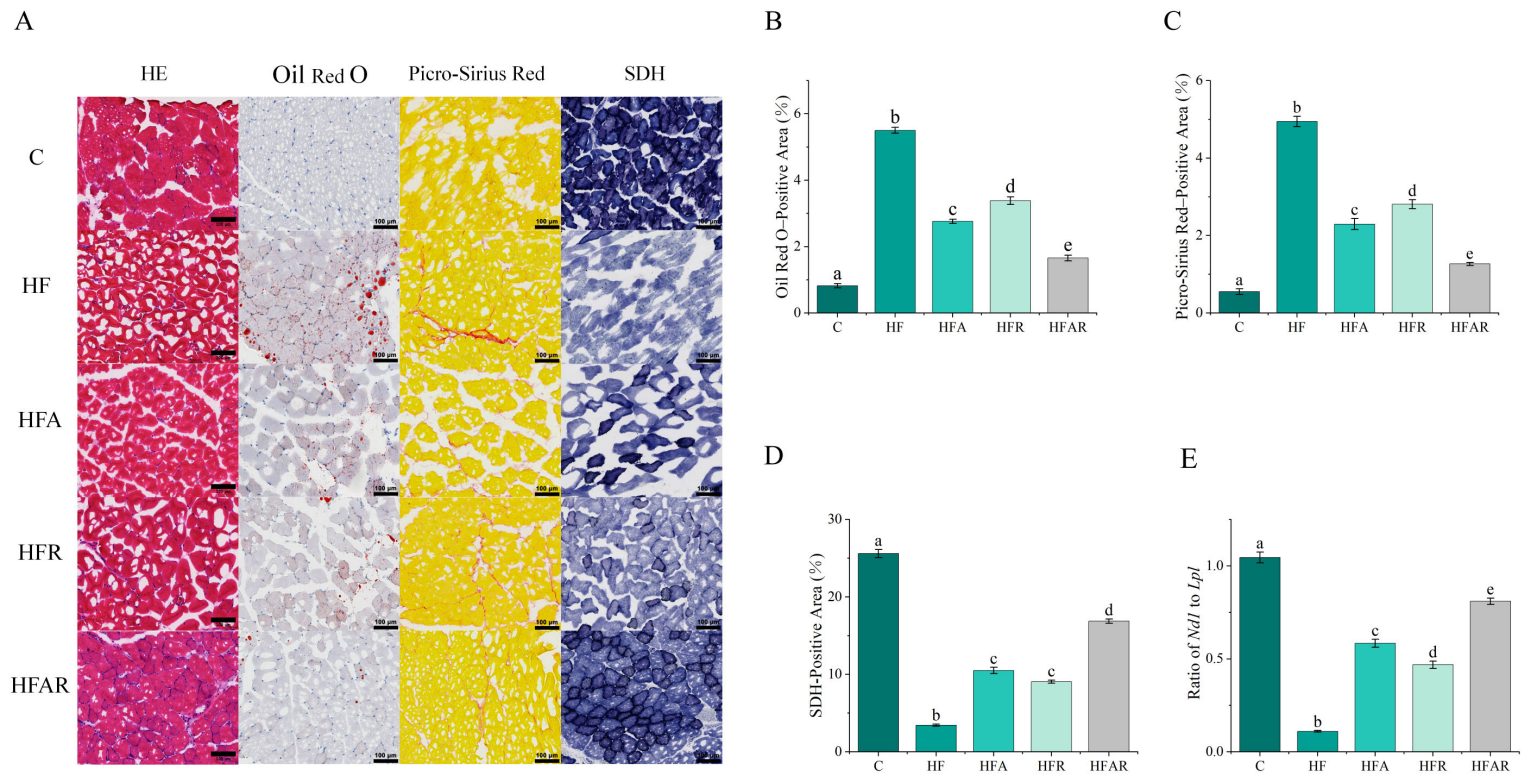
Effects of different exercise modalities on skeletal muscle histomorphology and mtDNA content in HFD-fed mice. **(A)** Representative images of HE staining (scale bar: 100 μm), Oil Red O staining (scale bar: 100 μm), Picro-Sirius Red staining (scale bar: 100 μm), and SDH staining (scale bar: 100 μm) of the tibialis anterior muscle. **(B)** Percentage of Oil Red O-positive area. **(C)** Percentage of Picro-Sirius Red-positive area. **(D)** Percentage of SDH-positive area. **(E)** mtDNA content (Nd1/Lpl ratio).Data are presented as mean ± SEM (n = 6 per group). Different letters indicate statistically significant differences among groups.

HE staining of skeletal muscle sections from all groups (**Figure 3A**) demonstrated marked pathological changes in the HF group. These changes were characterized primarily by the presence of centrally located nuclei in some fibers, and inflammatory cell infiltration. Histomorphological improvement was observed following intervention with different exercise modalities. Muscle morphology in the HFAR group most closely approximated that of the C group.

Oil Red O staining results (**Figures 3A, B**) indicated that the proportion of positively stained areas in the HF group was significantly higher than that in the C group (*p* < 0.01). All exercise groups exhibited significantly lower percentages of positively stained areas compared with the HF group (*p* < 0.01). Significant differences were observed among the exercise groups (*p* < 0.01). The HFAR group demonstrated the lowest percentage among the exercise groups; however, this value remained significantly higher than that of the C group (*p* < 0.01).

Picro-Sirius Red staining (**Figures 3A, C**) demonstrated that the extent of skeletal muscle fibrosis in the HF group was significantly greater than that in the C group (*p* < 0.01). All exercise groups exhibited a significantly lower degree of fibrosis compared with the HF group (*p* < 0.01); however, fibrosis remained significantly higher than that observed in the C group (*p* < 0.01). The degree of fibrosis in the HFAR group was significantly lower than that observed in both the HFA and HFR groups (*p* < 0.05), whereas no significant difference was found between the HFA and HFR groups.

SDH staining was conducted to assess the proportion of slow-twitch muscle fibers. The results (**Figures 3A, D**) demonstrated that the percentage of SDH-positive area in the HF group was significantly lower than that in the C group (*p* < 0.01). All exercise groups presented a significantly higher percentage of positive area compared with the HF group (*p* < 0.01); however, these values remained significantly lower than those in the C group (*p* < 0.01). Among the exercise groups, the HFAR group exhibited a significantly higher percentage of positive area than both the HFA and HFR groups (*p* < 0.01), with no significant difference observed between the latter two groups.

### 3.3 Effects on skeletal muscle mtDNA content

To further examine the effects of different exercise modalities on skeletal muscle mitochondrial biogenesis in obese mice, mtDNA copy number was quantified using RT-qPCR. The results (**Figure 3E**) demonstrated significant differences among the five groups (*p* < 0.01). Specifically, mtDNA content in the C group was significantly higher than that in all other groups, whereas the HF group exhibited the lowest level. Among the three exercise intervention groups, mtDNA content ranked from highest to lowest as follows: HFAR, HFA, and HFR. Given that mtDNA copy number serves as a surrogate marker of mitochondrial biogenesis rather than a direct measure of mitochondrial function, these findings suggest enhanced mitochondrial biogenesis in the exercise groups, particularly HFAR.

### 3.4 Effects of exercise interventions on gut microbiota in HFD-fed mice

**Figure 4:**
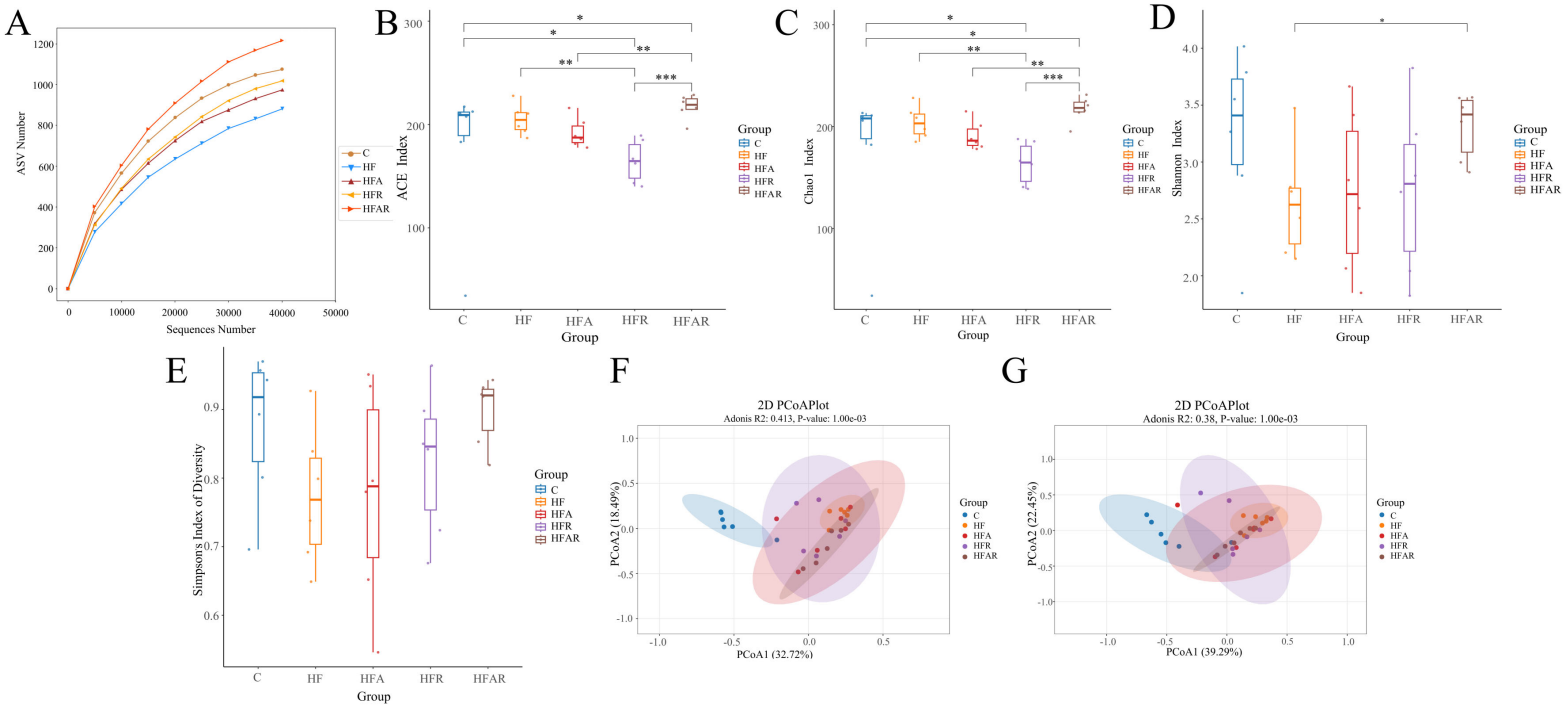
Effects of different exercise modalities on gut microbiota diversity. **(A)** Rarefaction curve; **(B)** ACE index; **(C)** Chao1 index; **(D)** Shannon index; **(E)** Simpson index of diversity; **(F)** PCoA based on Bray–Curtis distance; and **(G)** PCoA based on weighted UniFrac distance (*p < 0.05, **p < 0.01, ***p < 0.001).

To determine whether the sequencing depth was sufficient to accurately represent gut microbial diversity, rarefaction curve analysis was performed. A subset of sequences was randomly selected from each sample, and the corresponding number of species, represented by ASV counts, was calculated. Rarefaction curves were generated by plotting the number of sequences sampled against the number of observed species. As presented in **Figure 4A**, the curves for all groups approached a plateau when sequencing depth reached approximately 40,000 reads, indicating that the sequencing depth was adequate to capture the majority of microbial species present in the samples. These findings support the reliability of subsequent community diversity analyses.

### 3.5 Alpha diversity

Alpha (α) diversity of the intestinal microbiota was comprehensively assessed using the ACE, Chao1, Shannon, and Simpson indices. The ACE and Chao1 indices primarily reflect species richness, whereas the Shannon and Simpson indices assess species diversity and evenness within the microbial community. As presented in **Figure 4B** and C, significant differences in the ACE index were observed among the groups. When compared to the C group, the HFR group exhibited a significant decrease in the ACE index, whereas the HFAR group demonstrated a significant increase (*p* < 0.05). Compared with the HF group, a significant reduction in the ACE index was observed only in the HFR group (*p* < 0.01). Among the three exercise intervention groups, the ACE index in the HFAR group was significantly higher than that in both the HFA group (*p* < 0.01) and the HFR group (*p* < 0.001), while no significant difference was found between the HFA and HFR groups. The Chao1 index presented a highly consistent pattern of significant differences with the ACE index.

In contrast, different patterns were observed for the Shannon and Simpson indices. As presented in **Figures 4D, E**, a significant difference in the Shannon index was found only between the HF and HFAR groups (*p* < 0.05). The HF group exhibited the lowest Shannon index among all groups, whereas the indices in the exercise intervention groups (HFA, HFR, and HFAR) were restored to levels comparable to those of the C group. Notably, statistical significance was observed only between the HF and HFAR groups (*p* < 0.05), with no significant differences among the remaining groups. These findings indicate that exercise, particularly combined exercise, was associated with attenuation of high-fat diet–induced reductions in microbial diversity. However, changes in overall microbial community structure were still observed. Although the Simpson index demonstrated a trend like that of the Shannon index across groups, the differences did not reach statistical significance, potentially due to variability within groups.

### 3.6 Beta diversity

To comprehensively assess β-diversity of the intestinal microbiota, analyses were conducted based on Bray–Curtis and weighted UniFrac distance metrics. As presented in **Figure 4F**, PCoA based on Bray–Curtis distance (Adonis, R² = 0.413, *p* = 0.001) demonstrated significant overall differences in gut microbiota structure among the groups. The C group clustered in the left region of the coordinate space and was clearly separated from the other four groups. The HF group was distributed on the right side. The HFA, HFR, and HFAR groups were positioned between the C and HF groups, with partial overlap observed among them. Similarly, PCoA based on weighted UniFrac distance (Adonis, R² = 0.380, *p* = 0.001) confirmed significant differences in microbiota structure among the groups (**Figure 4G**). The separation between the C and HF groups was more pronounced, and the three exercise intervention groups were located closer to the C group along the PC1 axis. These findings indicate that a high-fat diet significantly altered the overall structure of the gut microbiota. Exercise interventions were associated with shifts in microbiota composition toward a profile more closely resembling that of the control group.

### 3.7 Gut microbiota composition

**Figure 5:**
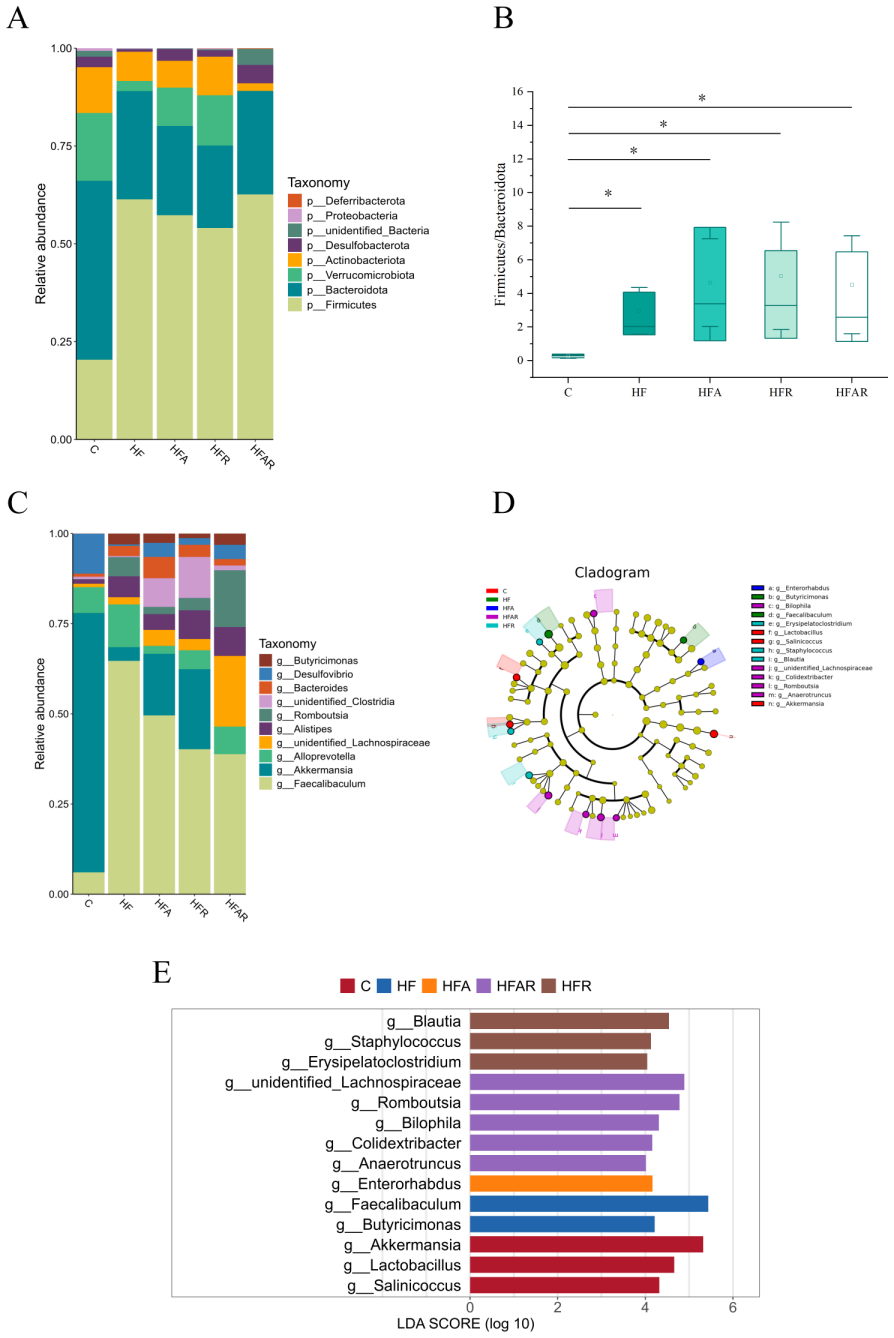
Effects of different interventions on gut microbiota composition and structure in the experimental model. **(A)** Relative abundance of microbial communities at the phylum level; **(B)** Firmicutes/Bacteroidota ratio (*p < 0.005); **(C)** relative abundance of microbial communities at the genus level; and **(D, E)** identification of differentially abundant taxa among groups based on LEfSe analysis.

To examine the effects of a high-fat diet and different exercise modalities on gut microbiota composition in mice, the relative abundances of major microbial taxa were analyzed. At the phylum level (**Figure 5A**), the gut microbiota across all groups was predominantly composed of Bacteroidota and Firmicutes. A significant difference in the Firmicutes/Bacteroidota (F/B) ratio was observed among the five groups (Kruskal–Wallis H test, H = 14.151, *p* = 0.007). *Post hoc* pairwise comparisons (Mann–Whitney U test with Bonferroni correction, *p* < 0.005) indicated that the F/B ratio in the C group was significantly lower than that in all other groups (*p* < 0.005). No significant differences in the F/B ratio were found among the four treatment groups (HF, HFA, HFR, and HFAR) (**Figure 5B**).

At the genus level (**Figure 5C**), the dominant genera in the C group included *Akkermansia*, *Desulfovibrio*, *Alloprevotella*, and *Faecalibaculum*. In the HF group, *Faecalibaculum* exhibited the highest relative abundance. The relative abundance of *Akkermansia* was markedly reduced in this group. Following exercise interventions, *Faecalibaculum* remained the most abundant genus in all exercise groups; however, its relative abundance decreased to varying degrees compared with the HF group.

In the HFA and HFR groups, the abundance of *Akkermansia* was partially restored and became the second most dominant genus. Additionally, certain genera with low relative abundance in the C and HF groups increased following exercise intervention. For example, unidentified_Clostridia appeared among the dominant genera in the HFA and HFR groups. In the HFAR group, the relative abundances of unidentified_Lachnospiraceae and *Romboutsia* increased substantially. These genera became the second and third most dominant genera, respectively.

### 3.8 Analysis of species differences between groups

Linear discriminant analysis effect size (LEfSe) analysis was performed to identify microbial taxa with significantly different abundances among groups (**Figures 5D, E**). With a linear discriminant analysis score > 4 as the threshold, 14 genera across five taxonomic groups were identified as significantly different at the genus level. These genera included *Salinicoccus*, *Lactobacillus*, *Akkermansia*, *Butyricimonas*, *Faecalibaculum*, *Enterorhabdus*, *Anaerotruncus*, *Colidextribacter*, *Bilophila*, *Romboutsia*, unidentified_Lachnospiraceae, *Erysipelatoclostridium*, *Staphylococcus*, and *Blautia*.

These taxa exhibited distinct abundance patterns across the five experimental groups. The findings indicate that different exercise modalities were associated with specific changes in gut microbiota composition. These changes may contribute to the modulation of host metabolic status.

### 3.9 Effects on serum differential metabolites

**Figure 6:**
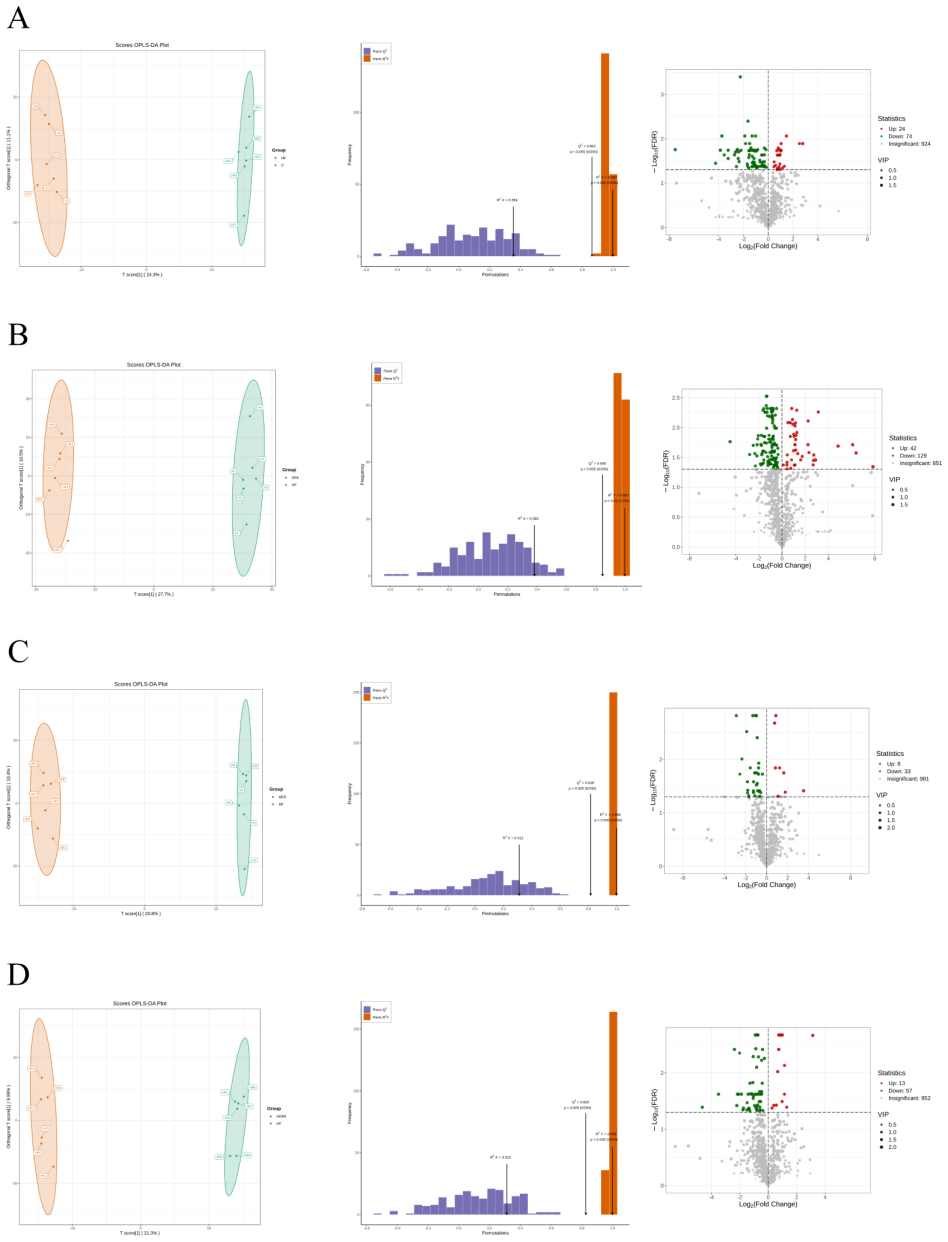
Differential metabolites between groups. **(A)** C vs HF; **(B)** HF vs HFA; **(C)** HF vs HFR; and **(D)** HF vs HFAR.

**Figure 7:**
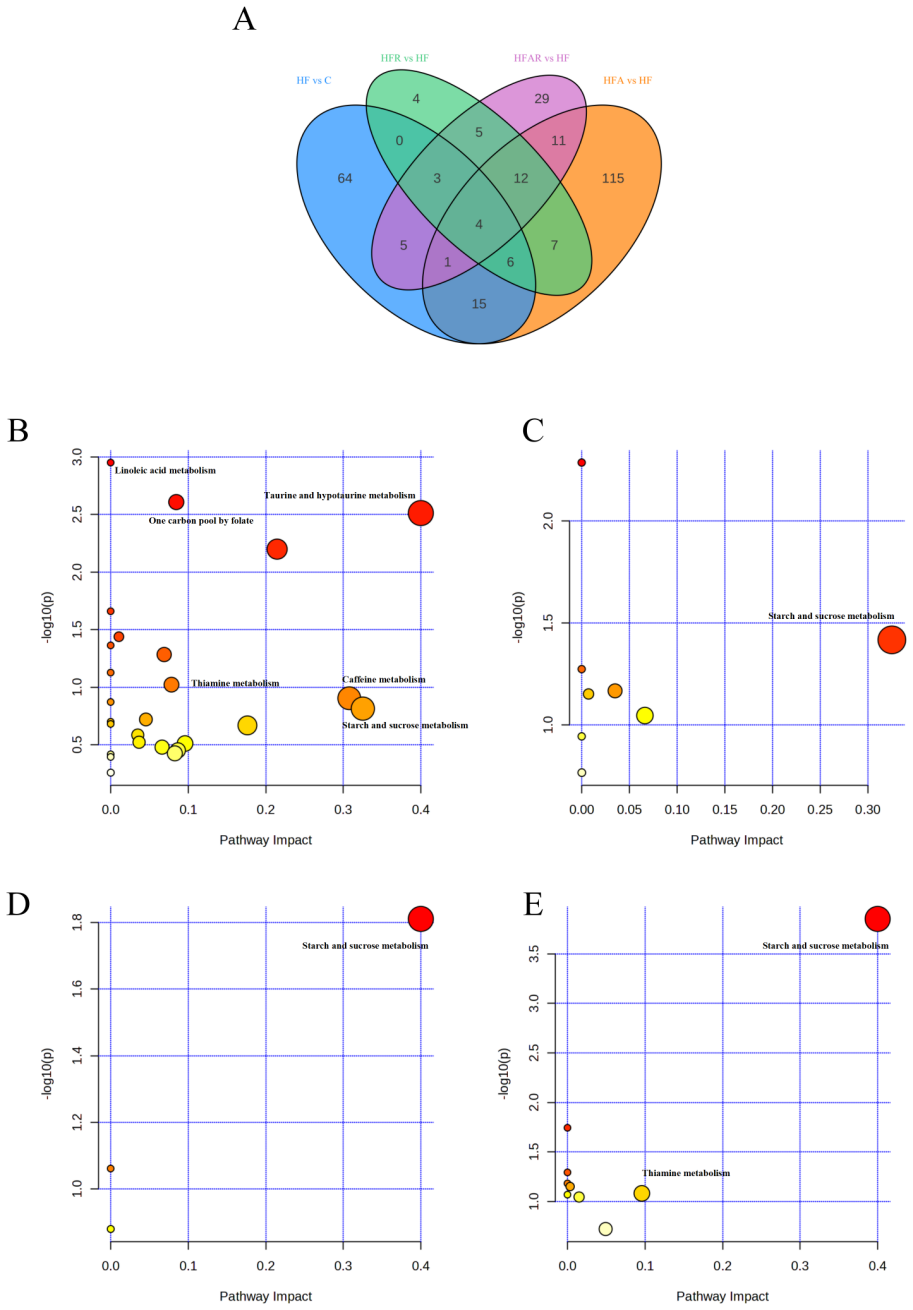
Analysis of differential metabolites and metabolic pathways based on metabolomics. **(A)** Venn diagram; **(B)** metabolic pathway analysis of C vs HF; **(C)** metabolic pathway analysis of HF vs HFA; **(D)** metabolic pathway analysis of HF vs HFR; and **(E)** metabolic pathway analysis of HF vs HFAR.

Untargeted metabolomic analysis was conducted on serum samples from the five groups. OPLS-DA demonstrated clear separation between the C and HF groups (**Figure 6A**, Q² = 0.863, R²Y = 0.997). Clear separation was also observed between the HF group and the HFA (**Figure 6B**, Q² = 0.846, R²Y = 0.997), HFR (**Figure 6C**, Q² = 0.816, R²Y = 0.998), and HFAR groups (**Figure 6D**, Q² = 0.826, R²Y = 0.998), with *p* < 0.005. Permutation testing confirming the robustness of the model (**Figures 6**, **7**). From the OPLS-DA results, differential metabolites were identified using the criteria of VIP > 1, *p* < 0.05, and fold change ≥ 1.2 or ≤ 0.83. A total of 278 differential metabolites were found. Between the C and HF groups, 96 significantly altered metabolites were identified, including 72 downregulated and 24 upregulated in the HF group (**Figure 6A**).

Comparison between the HF and HFA groups indicated 170 differential metabolites, of which 129 were downregulated and 41 upregulated in the HFA group (**Figure 6B**). Between the HF and HFR groups, 41 differential metabolites were identified, including 33 downregulated and 8 upregulated in the HFR group (**Figure 6C**). Finally, 69 differential metabolites were identified between the HF and HFAR groups, with 57 downregulated and 12 upregulated in the HFAR group (**Figure 6D**).

Compared with the C group, serum levels of Tagatose, Spermidine, Allantoic acid, Phenazopyridine, Dehydrocostus lactone, Cholic acid, p-Cresol, Mannitol 1-phosphate, Glucose, and 5-Acetamidovalerate were significantly increased in the HF group (*p* < 0.05), whereas levels of Firefly luciferin, Glyphosate, and 3-Methylxanthine were significantly decreased (*p* < 0.05). Different exercise interventions partially reversed these changes in specific metabolites (*p* < 0.05). The identified metabolites were classified into the following categories: alkaloids; organic acids and their derivatives; heterocyclic compounds; bile acids; nucleotides and their metabolites; benzene and substituted derivatives; carbohydrates and their metabolites; and amino acids and their metabolites. The identities, VIP, fold-change trends, and FDR for each contrast are summarized in **Table 2**. Pathway analysis of the differential metabolites was conducted using MetaboAnalyst 6.0 (www.metaboanalyst.ca). Compared with the C group, seven metabolic pathways were significantly altered in the HF group (**Figure 7B**), among which taurine and hypotaurine metabolism and glycerophospholipid metabolism demonstrated higher impact scores. Distinct regulatory patterns were observed across exercise interventions. In the HFAR group, taurine and hypotaurine metabolism and thiamine metabolism were significantly restored (**Figure 7E**). In the HFR group, only taurine and hypotaurine metabolism was significantly altered (**Figure 7D**), whereas in the HFA group, starch and sucrose metabolism was significantly affected (**Figure 5C**).

**Table 2:** Differential metabolites identified through cross-comparison among the different groups.

Compounds	Class	C vs HF				HF vs HFA				HF vs HFR				HF vs HFAR			
		VIP	FC	Trend	FDR	VIP	FC	Trend	FDR	VIP	FC	Trend	FDR	VIP	FC	Trend	FDR
Spermidine	Alkaloids	1.62	1.66	↑	0.02	1.64	0.55	↓	0.01	—	—	—	—	—	—	—	—
5-Acetamidovalerate	Organic acid and Its derivatives	1.38	2.23	↑	0.04	1.45	0.48	↓	0.03	—	—	—	—	—	—	—	—
Allantoic acid		1.78	2.05	↑	0.02	1.59	0.41	↓	0.01	1.95	0.46	↓	0.02	—	—	—	—
Phenazopyridine	Heterocyclic compounds	1.51	1.66	↑	0.04	1.50	0.64	↓	0.04	—	—	—	—	—	—	—	—
Dehydrocostus lactone		1.52	2.51	↑	0.01	1.46	0.65	↓	0.04	—	—	—	—	—	—	—	—
Firefly luciferin		1.64	0.74	↓	0.03	—	—	—	—	2.06	1.82	↑	0.01	2.04	1.82	↑	0.01
Cholic acid	Bile acids	1.89	2.78	↑	0.01	1.57	0.48	↓	0.02	1.84	0.41	↓	0.03	—	—	—	—
3-Methylxanthine	Nucleotide and Its metabolites	1.54	0.80	↓	0.04	1.04	1.35	↑	0.04	2.02	2.12	↑	0.04	2.08	1.97	↑	0.01
p-Cresol	Benzene and substituted derivatives	1.74	1.46	↑	0.01	1.47	0.34	↓	0.01	1.80	0.27	↓	0.01	—	—	—	—
Mannitol 1-phosphate	Carbohydrates and Its metabolites	1.60	1.50	↑	0.04	1.51	0.49	↓	0.02	—	—	—	—	—	—	—	—
Glucose		1.59	1.39	↑	0.03	1.36	0.64	↓	0.04	—	—	—	—	—	—	—	—
Tagatose		1.62	1.93	↑	0.02	1.67	0.37	↓	0.01	—	—	—	—	—	—	—	—
Glyphosate	Amino acid and Its metabolites	1.59	0.79	↓	0.04	—	—	—	—	2.07	1.68	↑	0.01	2.02	1.67	↑	0.01

↑ = increased, ↓ = decreased, – = not significant/not detected in that contrast.

### 3.10 Correlation analysis among gut microbiota, serum metabolites, and body composition and skeletal muscle functional indices

After identifying differential gut microbial genera and serum metabolites following different exercise interventions in HFD-fed obese mice, Spearman rank correlation analysis was used to evaluate associations between differential genera and differential metabolites, between differential genera and body composition and skeletal muscle functional indices, and between differential metabolites and body composition and skeletal muscle functional indices.

**Figure 8:**
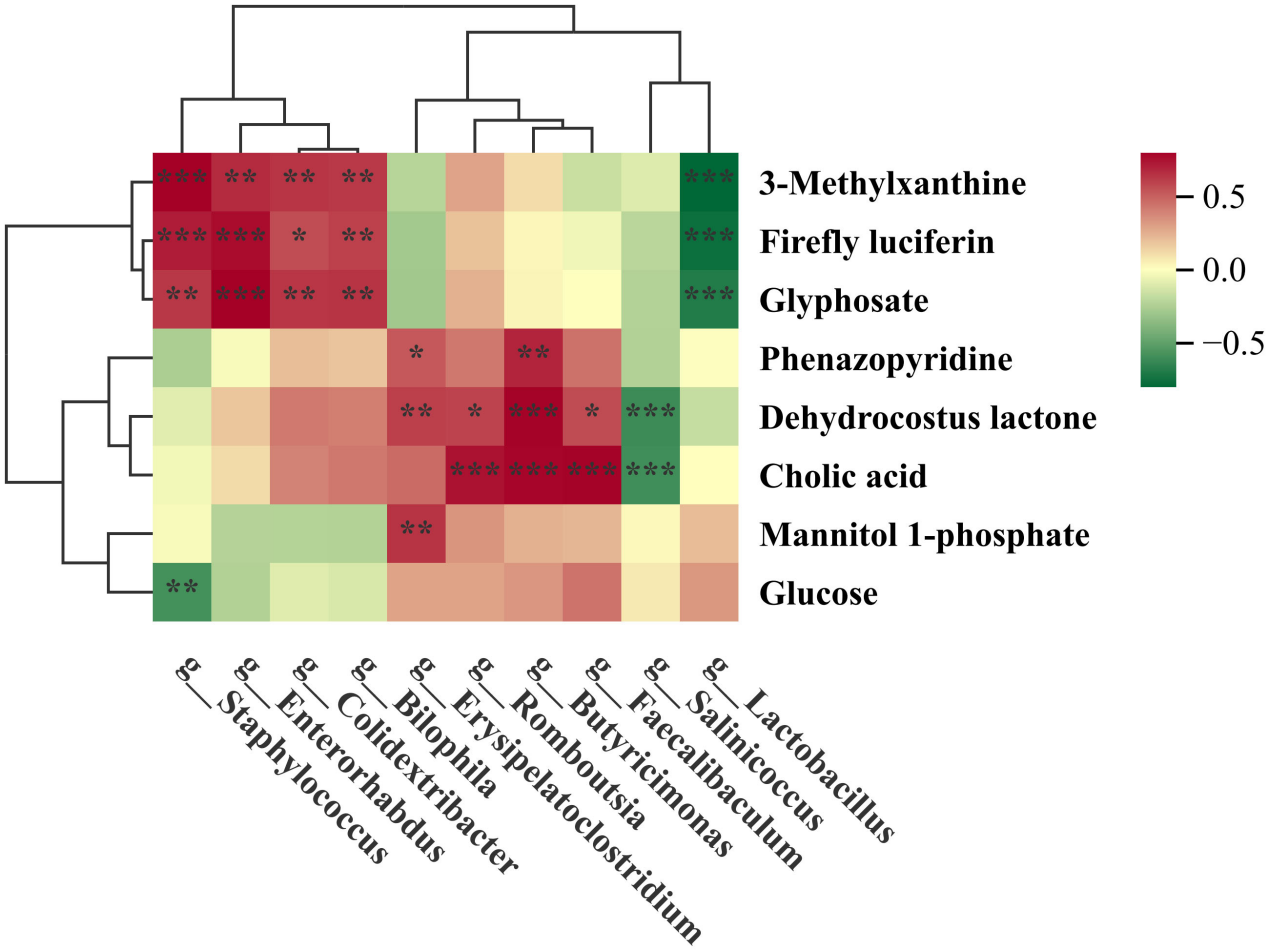
Correlation heatmap between differentially abundant bacterial genera and differential metabolites. *p< 0.05, **p< 0.01, ***p< 0.001.

In the correlation analysis between differential genera and metabolites (**Figure 8**), Lactobacillus was negatively correlated with 3-Methylxanthine, Firefly luciferin, and Glyphosate. Cholic acid was positively correlated with Faecalibaculum, Butyricimonas, and Romboutsia, and negatively correlated with Salinicoccus. Dehydrocostus lactone was positively correlated with Butyricimonas and strongly negatively correlated with Salinicoccus. 3-Methylxanthine was positively correlated with Staphylococcus, Enterorhabdus, and Colidextribacter. Glucose was negatively correlated with Staphylococcus.

**Figure 9:**
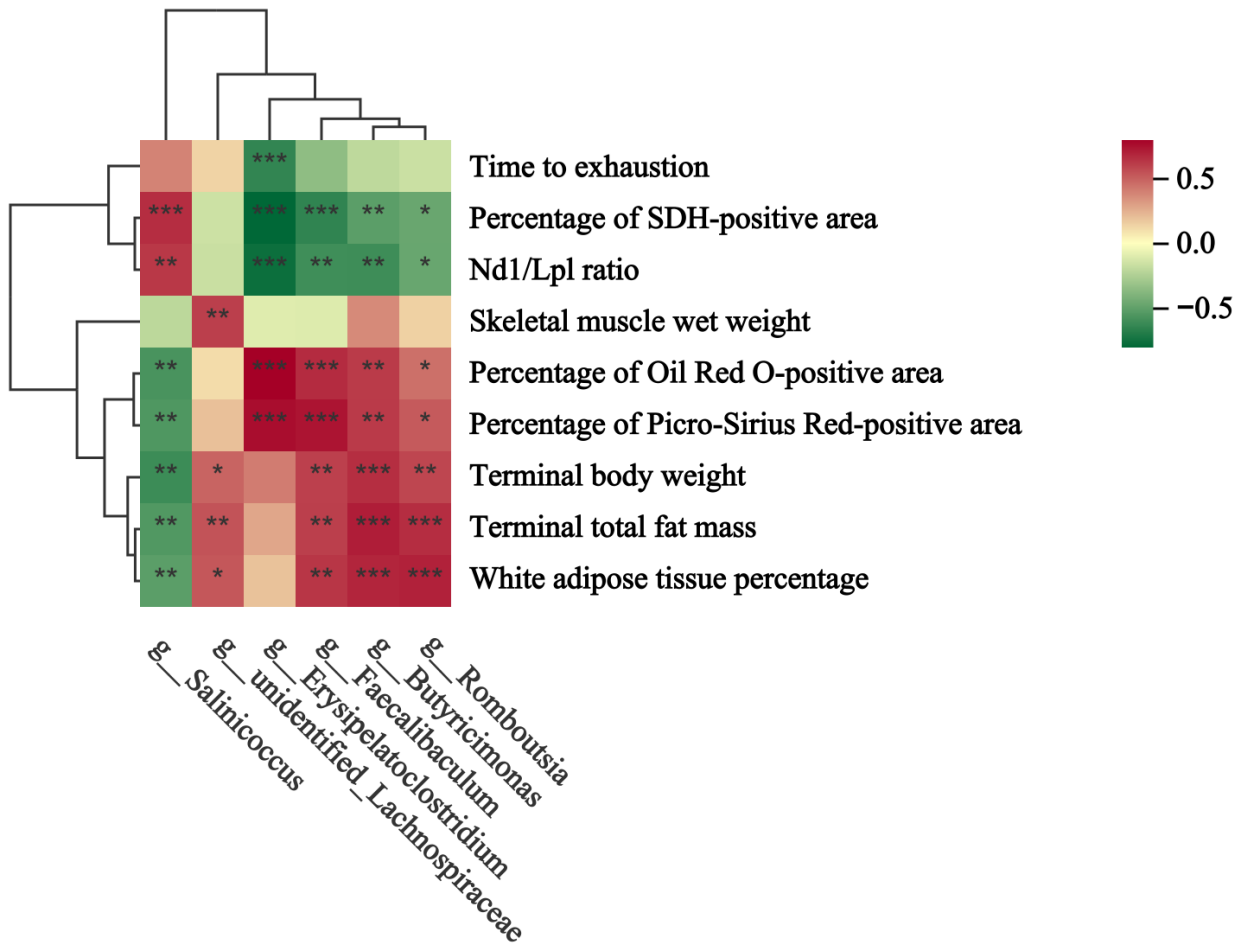
Correlation heatmap between differentially abundant bacterial genera and phenotypic indices. *p< 0.05, **p< 0.01, ***p< 0.001.

In the correlation analysis between differential genera and body composition and skeletal muscle functional indices (**Figure 9**), Erysipelatoclostridium was positively correlated with Oil Red O-positive area and Picro-Sirius Red-positive area, and negatively correlated with SDH-positive area and Nd1/Lpl ratio, whereas Salinicoccus displayed the opposite pattern. Butyricimonas was positively correlated with white adipose tissue percentage and terminal total fat mass. Faecalibaculum was positively correlated with Picro-Sirius Red-positive area. Romboutsia was positively correlated with white adipose tissue percentage and terminal total fat mass.

**Figure 10:**
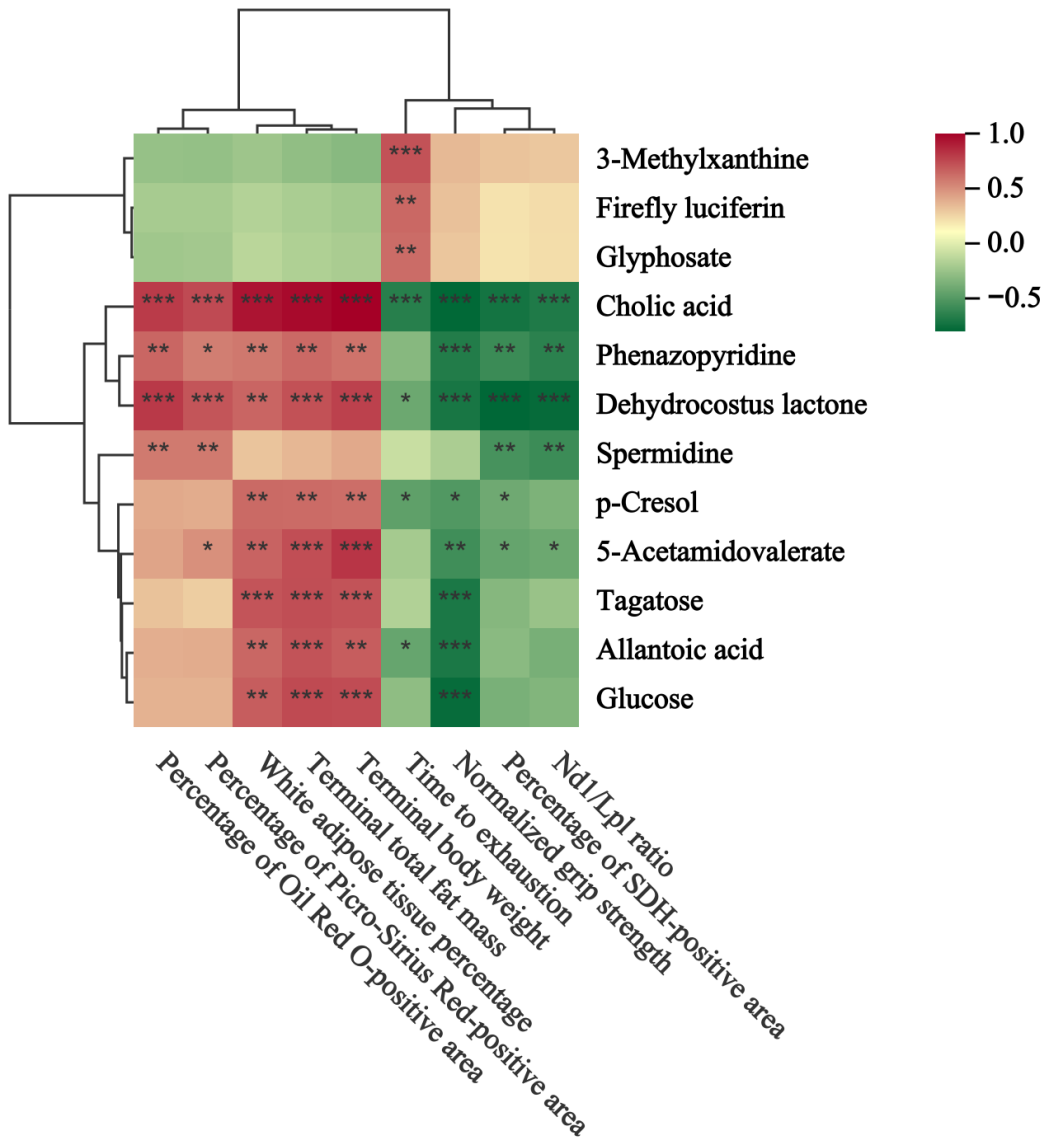
Correlation heatmap between differential metabolites and phenotypic indices. *p< 0.05, **p< 0.01, ***p< 0.001.

In the correlation analysis between differential metabolites and body composition and skeletal muscle functional indices (**Figure 10**), Cholic acid was positively correlated with white adipose tissue percentage, terminal total fat mass, and terminal body weight, and negatively correlated with normalized grip strength and Nd1/Lpl ratio. Dehydrocostus lactone was positively correlated with white adipose tissue percentage and terminal total fat mass, and negatively correlated with Nd1/Lpl ratio and normalized grip strength. 3-Methylxanthine was positively correlated with exhaustion time. Glucose was negatively correlated with normalized grip strength and positively correlated with white adipose tissue percentage.

### 3.11 Mediation analysis

Based on the correlation analysis results, exploratory mediation analyses were conducted with differential genera as independent variables (X), differential metabolites as mediators (M), and relevant phenotypic indices as dependent variables (Y). In the HFAR group, the indirect effect of “Colidextribacter → 3-Methylxanthine → Time to exhaustion” was significant. Colidextribacter abundance was positively associated with 3-Methylxanthine, and 3-Methylxanthine was positively associated with Time to exhaustion. After inclusion of the mediator, the direct effect was substantially attenuated, suggesting that 3-Methylxanthine partially mediated the promoting effect of this genus on endurance performance. In the HFA group, the indirect effect of “Enterorhabdus → 3-Methylxanthine → Time to exhaustion” was marginally significant. The lower bound of its bootstrap confidence interval was positive, but p = 0.118, indicating that the statistical support for this pathway remains tentative and requires validation in larger samples. In the HFR group, the indirect effect of “Butyricimonas → Cholic acid → White adipose tissue percentage” was not significant.

In summary, both Colidextribacter in the HFAR group and Enterorhabdus in the HFA group influenced Time to exhaustion through 3-Methylxanthine, with the former receiving significant statistical support and the latter being marginally significant. The convergence of these two pathways on the same metabolite suggests that different exercise modalities may enrich distinct microbial genera that act through a shared purine metabolic pathway to influence skeletal muscle endurance.

## 4 Discussion

Exercise represents a defined physiological stimulus that induces adaptive responses affecting systemic metabolism and skeletal muscle function. In the present study of exercise interventions in high-fat diet–induced obese mice, distinct exercise modalities were associated with varying degrees of reduction in white adipose tissue percentage, with resistance exercise demonstrating the most pronounced effect. Consistently, the HFR group exhibited significantly lower terminal fat mass and terminal body weight compared with the HF group. The energy intake data, however, revealed that the HFR group maintained very high caloric intake throughout the intervention, second only to the sedentary HF group. The co-occurrence of high energy intake and significant fat loss argues against the simplistic explanation that the metabolic benefits of resistance exercise are solely mediated by appetite suppression; rather, increased energy expenditure and favorable nutrient partitioning toward lean tissue likely play a more prominent role (20). Regarding skeletal muscle endurance and strength, all exercise modalities effectively attenuated obesity-associated functional decline; however, no significant differences were observed among the exercise types. Although absolute muscle wet weight did not differ significantly among groups, relative muscle weight normalized to terminal body weight showed a trend toward restoration in exercise groups. This was paralleled by a dissociation between absolute and relative grip strength—the former unaltered across groups, the latter significantly impaired in the HF group and rescued by exercise—suggesting that exercise enhanced “muscle quality” (force output and metabolic capacity per unit body mass) rather than absolute muscle mass. Notably, combined exercise (HFAR group) was associated with greater improvements in muscle histomorphology, increased gut microbiota diversity, and enhanced skeletal muscle mitochondrial biogenesis. The observed variation among these outcome measures suggests that exercise exerts modality-specific effects on skeletal muscle, which may be more prominently reflected at structural and metabolic levels than in functional performance indices. The underlying mechanisms require further investigation through integrated analyses of gut microbiota and metabolomic profiles.

Different exercise modalities were associated with significant attenuation of obesity-induced pathological changes in skeletal muscle morphology. This was reflected by the amelioration of pathological morphological alterations observed on HE staining, reduced lipid accumulation on Oil Red O staining, and decreased skeletal muscle fibrosis on Picro-Sirius Red staining across all exercise intervention groups. Previous studies have demonstrated that aerobic exercise can reduce lipid deposition by modulating the expression of genes involved in lipid metabolism, thereby indirectly alleviating muscle fiber atrophy (21). In addition, aerobic exercise has been reported to reduce skeletal muscle fibrosis through suppression of *Ccn2* gene expression (22). Resistance exercise promotes skeletal muscle hypertrophy and increases muscle fiber cross-sectional area. Concurrently, it significantly downregulates pro-inflammatory factors while upregulating anti-inflammatory factors, contributing to reduced skeletal muscle fibrosis (20).

Combined exercise may incorporate the benefits of both modalities, potentially generating synergistic effects in improving muscle fiber structure, regulating lipid metabolism, and suppressing inflammatory responses, thereby more comprehensively attenuating obesity-induced pathological remodeling of skeletal muscle. Notably, although the HFAR group demonstrated the most pronounced improvements at the levels of tissue morphology and energy metabolism, corresponding enhancements in skeletal muscle functional indices were not observed. This discrepancy may be attributable to potential interference effects of combined exercise on functional recovery. A study involving high-level athletes reported that isolated resistance training was superior to combined training in enhancing muscle strength (23). Evidence suggests that improvements in skeletal muscle strength depend on both muscle hypertrophy and neural adaptation, with neural adaptations typically occurring later than morphological changes (24). These findings indicate that functional improvement may require a longer intervention period. Therefore, the absence of significant functional enhancement in the HFAR group may reflect limitations related to intervention duration rather than a lack of efficacy of combined exercise.

Furthermore, the oxidative stress and inflammation induced by HFD-associated obesity may render the physiological effects of exercise on skeletal muscle more complex. Under the persistent metabolic burden of a high-fat diet, acute or high-intensity exercise may transiently exacerbate oxidative stress and skeletal muscle microdamage (25). However, the protocol employed in this study—consisting of a 1-week adaptation period followed by 8 weeks of progressive training—likely permitted the gradual establishment of chronic protective adaptations, ultimately yielding positive remodeling of skeletal muscle morphology and function at the study endpoint.

A HFD promotes systemic lipid accumulation and disrupts gut microbiota homeostasis (26). According to evidence, skeletal muscle structure and function are influenced by the composition and abundance of the gut microbiota, a relationship described as the “gut microbiota–muscle axis” (27). This interaction is mediated, in part, through microbiota-derived metabolites that modulate skeletal muscle function (10). In this study, consumption of a HFD was associated with a significant reduction in α-diversity and a marked shift in β-diversity of the gut microbiota. In addition, the Firmicutes/Bacteroidota ratio increased significantly, consistent with previous reports describing high-fat diet–induced gut dysbiosis (28, 29).

Following exercise interventions, gut microbial species diversity was restored to varying extents, with the HFAR group demonstrating the most pronounced recovery. These findings indicate that combined exercise modalities may enhance gut ecosystem stability. Notably, the Firmicutes/Bacteroidota ratio did not significantly decrease in any exercise group and remained elevated. This may reflect modality-specific effects of exercise on microbial community restructuring, consistent with the observed separation in β-diversity among exercise groups and the variation in dominant genera beyond Firmicutes. LEfSe further demonstrated exercise modality–specific changes in gut microbiota composition.

Enrichment of *Enterorhabdus* in the HFA group may contribute to improvements in obesity and associated skeletal muscle dysfunction through enhanced short-chain fatty acid production and modulation of genes involved in glucose and lipid metabolism (30, 31). Significant enrichment of *Blautia* in the HFR group was associated with regulation of lipid metabolism and suppression of chronic inflammation, corresponding to the significant reduction in white adipose tissue percentage observed in this group (32, 33).

In the HFAR group, enrichment of multiple genera indicated potential synergistic effects on skeletal muscle function through diverse mechanisms. For example, *Colidextribacter* and *Romboutsia* may enhance skeletal muscle antioxidant capacity and glucose metabolism. Additionally, unidentified_Lachnospiraceae (belonging to the Lachnospiraceae family) may support muscle function through production of anti-inflammatory metabolites and improved energy metabolism (34).

The effects of gut microbiota on host skeletal muscle are mediated in part by microbiota-derived metabolites that enter the systemic circulation and subsequently reach muscle tissue. Accordingly, serum metabolomic profiles were further analyzed. Among metabolites known to be associated with skeletal muscle function, the significant alterations observed in the HF group compared with the C group indicate substantial metabolic disturbances in the HF group, which may contribute to adverse changes in skeletal muscle structure and function.

For example, elevated Cholic acid is associated with bile acid metabolism disorders and, in this context, may contribute to skeletal muscle atrophy and reduced strength, potentially through activation of the membrane receptor TGR5 (35). In addition, reduced levels of 3-Methylxanthine, a methylxanthine compound, may decrease activation of ryanodine receptors, thereby impairing muscle fiber contractile function (36).

Exercise interventions partially reversed these metabolite alterations, although the extent of reversal varied across modalities. Notably, Glyphosate, which has been reported to be inversely associated with skeletal muscle health, was significantly increased in the HFR and HFAR groups (37). This increase may be related to enhanced adipose tissue mobilization during exercise. This may result in the release of lipophilic environmental pollutants such as Glyphosate from adipose depots into the circulation (38). These findings indicate that although exercise improves metabolic disturbances, redistribution of environmental toxicants may occur. Distinct exercise modalities demonstrated specificity in metabolic pathway regulation. Both resistance and combined exercise significantly influenced taurine and hypotaurine metabolism, which may support skeletal muscle health through anti-inflammatory, antioxidant, and metabolic mechanisms. Combined exercise additionally regulated thiamine metabolism, which may contribute to preservation of skeletal muscle function by mitigating lipid metabolism disturbances and reducing production of pro-inflammatory mediators. In contrast, aerobic exercise specifically modulated starch and sucrose metabolism, thereby influencing skeletal muscle energy homeostasis. Collectively, these findings underscore the modality-specific metabolic effects of exercise interventions.

Spearman’s correlation analysis demonstrated a complex interaction network between gut microbiota, serum metabolites, and body composition and skeletal muscle functional indices under conditions of a HFD and following exercise interventions. In high-fat diet–induced obesity, Cholic acid levels were significantly elevated and showed strong positive correlations with the enriched genera *Faecalibaculum* and *Butyricimonas*, as well as with white adipose tissue percentage, terminal total fat mass, and terminal body weight, and negative correlations with relative grip strength and mtDNA copy number. These findings indicate an association between high-fat diet–associated proliferation of specific bacterial taxa and dysregulation of host bile acid metabolism, the obese phenotype, and impaired skeletal muscle function. In contrast, the negative correlation between Cholic acid and *Salinicoccus* supports a potential association between this genus and maintenance of bile acid metabolic balance under physiological conditions.

In addition, *Lactobacillus* exhibited a negative correlation with 3-Methylxanthine. Prior studies have reported a significant reduction in *Lactobacillus* abundance following exercise training in individuals with obesity or type 2 diabetes. This may contribute to upregulation of the purine cycle, thereby improving muscle function and reducing lipid accumulation (39, 40). In the present study, the elevation of 3-Methylxanthine, a purine metabolite, across the exercise groups and its positive correlation with exhaustion time may reflect this process. Furthermore, the positive correlations of 3-Methylxanthine with Staphylococcus, Enterorhabdus, and Colidextribacter under different exercise interventions suggest that the modality-specific proliferation of these genera may be involved in the regulation of purine metabolism and influence host metabolic health (41).These cross-kingdom correlation results, spanning the gut microbiota, serum metabolites, and host skeletal muscle phenotypes, provided candidate pathways for the subsequent exploratory mediation analyses.

Based on the findings of the Spearman correlation analysis described above, exploratory mediation analyses were conducted to examine potential cascading effects within the candidate pathways. In the combined exercise (HFAR) group, Colidextribacter appeared to exert a positive indirect effect on Time to exhaustion, potentially through promoting the production of 3-Methylxanthine. In the aerobic exercise (HFA) group, Enterorhabdus established a marginally significant association with Time to exhaustion via the same metabolite. Elevated levels of 3-Methylxanthine may modulate ryanodine receptors activation, thereby influencing muscle fiber contractile function, and may also participate in the purine cycle to enhance muscle metabolic efficiency [36. 39]. The convergence of these two pathways—derived from different exercise modalities—on the same metabolite suggests that, although aerobic and combined exercise enrich distinct gut microbial genera, they may influence skeletal muscle endurance through a shared purine metabolic node. This also implies that 3-Methylxanthine may serve as a potential enteric-derived metabolic bridge through which exercise improves muscle function.

In contrast, although Butyricimonas—a genus enriched in the resistance exercise (HFR) group—exhibited a strong positive correlation with both cholic acid and white adipose tissue percentage, the pathway “Butyricimonas → Cholic acid → White adipose tissue percentage” was not supported by the mediation analysis. This null result likely reflects the practical constraints inherent to multi-omics studies rather than negating the biological role of cholic acid, namely the instability of indirect effect estimates in regression models with limited sample sizes (42).

In summary, different exercise interventions were associated with significant attenuation of high-fat diet–induced obesity and skeletal muscle dysfunction, with effects demonstrating modality-specific characteristics. This specificity was reflected not only in improvements in skeletal muscle morphology and function but also in changes of metabolic pathways and gut microbiota composition. The exploratory mediation analyses in this study provide preliminary evidence for microbiota-driven metabolic cascades within the gut-muscle axis: genera enriched by different exercise modalities were statistically associated with skeletal muscle endurance through 3-Methylxanthine, suggesting that purine metabolism may serve as a shared downstream pathway through which exercise improves muscle function. These findings support the potential of targeted exercise modalities as interventional strategies for obesity and related skeletal muscle impairment, offering a novel intervention approach for obesity and its associated skeletal muscle dysfunction. However, the precise mechanisms underlying the distinct effects of each exercise modality on obesity and skeletal muscle dysfunction remain to be further elucidated.

## 5 Conclusion

Aerobic exercise, resistance exercise, and combined aerobic–resistance exercise were each associated with improvements in obesity-induced skeletal muscle morphological alterations and functional impairments, although the magnitude of these effects varied among modalities. Aerobic exercise primarily enhanced metabolic quality, while resistance exercise promoted body composition rebalancing by reducing adiposity while preserving muscle mass, and combined exercise produced the most pronounced improvements in histomorphology and mitochondrial content. Distinct exercise modalities were associated with differential remodeling of gut microbiota composition and serum metabolomic profiles. Correlation and exploratory mediation analyses identified genus-metabolite-phenotype cascades converging on 3-methylxanthine, suggesting that distinct exercise modalities may influence skeletal muscle endurance through different microbial genera via shared purine metabolism. These findings suggest that the mechanisms through which various exercise modalities contribute to skeletal muscle improvement via the “gut microbiota–muscle axis” are not uniform and may involve distinct regulatory pathways.

This study has several limitations. Although the exercise protocols were adapted from established models, optimal exercise intensity was not systematically assessed, which may have limited the extent of functional improvement observed in skeletal muscle. The sample size was limited to six mice per group, with mediation models conducted on only twelve samples per comparison; moreover, gut microbiota, serum metabolites, and skeletal muscle phenotypes were all measured at a single terminal time point in a cross-sectional design, precluding causal inference, and the mediation results should therefore be interpreted as hypothesis-generating. Food intake was recorded at the cage level, preventing assessment of within-group variability. Muscle fiber cross-sectional area was not directly quantified, and mtDNA copy number served only as a surrogate marker of mitochondrial biogenesis without confirmatory functional assays such as citrate synthase activity or PGC1α expression. The use of chow-fed mice as the sole non-HFD control may confound microbiota comparisons due to differences in both macronutrient composition and energy density. Future research should employ longitudinal designs with larger sample sizes, systematically evaluate the dose-response effects of exercise intensity, and incorporate targeted metabolomics and functional validation experiments to confirm the candidate microbiota-metabolite pathways identified herein, thereby further clarifying the mechanisms through which distinct exercise modalities improve skeletal muscle function via the “gut microbiota–muscle axis”.

## Data Availability

The metabolomics data presented in this study are deposited in the MetaboLights repository under accession number MTBLS13878. Available at: https://www.ebi.ac.uk/metabolights/MTBLS13878. The gut microbiota 16S rRNA sequencing data presented in this study are deposited in the NCBI Sequence Read Archive (SRA) under BioProject accession number PRJNA1420840. Available at: https://www.ncbi.nlm.nih.gov/bioproject/PRJNA1420840.
